# Challenges of Diagnosing Viral Myocarditis in Adolescents in the Era of COVID-19 and MIS-C

**DOI:** 10.1155/2021/4797498

**Published:** 2021-10-04

**Authors:** Hemali P. Shah, Richard Frye, Sunny Chang, Erin Faherty, Jeremy Steele, Ruchika Karnik

**Affiliations:** Department of Pediatrics, Section of Pediatric Cardiology, Yale University School of Medicine, New Haven, CT, USA

## Abstract

Myocarditis has a wide array of clinical presentations ranging from asymptomatic to sudden cardiac death. Pediatric myocarditis is a rare disease, with an estimated annual incidence of 1 to 2 per 100,000 children though its true prevalence remains unknown due to its variable and often subclinical presentation. The diagnosis of myocarditis is challenging in the era of COVID-19 and Multisystem Inflammatory Syndrome in Children (MIS-C), which can have overlapping clinical conundrum. Here, we present a case of a 17-year-old male presenting with chest tightness, shortness of breath, and electrocardiogram (EKG) findings concerning for myocardial injury along with elevated inflammatory markers such as D-dimer, ESR (Erythrocyte Sedimentation Rate), and CRP (C-Reactive Protein). We discuss the key elements of our clinical experience with this case and review the literature for pediatric myocarditis, with a focus on differentiating it from MIS-C in the current COVID-19 pandemic era.

## 1. Introduction

COVID Myocarditis is a rare diagnosis in pediatrics and has a heterogeneous clinical course ranging from asymptomatic, gradual-onset congestive heart failure (CHF) to fulminant myocarditis complicated by cardiogenic shock and sudden death [1,2]. This range of presentations makes the diagnosis of myocarditis exceptionally challenging, especially in the pediatric population and in the current era of the COVID-19 pandemic. The possibility of COVID-19 and MIS-C with their own breadth of clinical presentations adds another layer of nuance to diagnosing myocarditis.

In children ultimately diagnosed with myocarditis, tachycardia, tachypnea, and an abnormal respiratory examination are among the most frequently reported presenting symptoms in the emergency department [2,3]. Other presenting symptoms include chest pain, syncope, palpitations, and isolated gastrointestinal symptoms (e.g., abdominal pain and vomiting). A pathological confirmation is required for definitive diagnosis of myocarditis. However, given the invasive nature of endomyocardial biopsies, cardiac MRI is currently the gold standard noninvasive modality for diagnosis [1].

Beyond varying symptomatology, the clinical outcomes of children with myocarditis are also widely variable. The overall mortality rate of myocarditis has been reported to be 7–15% in the pediatric population; of those that survive, many develop long-term sequelae such as dilated cardiomyopathy and CHF [1–4]. Furthermore, it is difficult to predict which children may have poor outcomes and are at risk for developing the aforementioned long-term sequelae; studies have not yet found definitive predictors for prognosis [1,2,4]. Given these consequences and prognostic uncertainty, pediatric myocarditis warrants timely assessment and supportive measures, which are the mainstay of treatment.

## 2. Case Presentation

A 17-year-old male with a history of asthma presented to an outside adult emergency department (ED) with 24 hours of progressively worsening shortness of breath and chest tightness at rest that was unresponsive to bronchodilators. No recent illness was reported. He had a dental cleaning visit 3 days prior. He had no significant family history of cardiac disease, sudden cardiac death, or coagulopathies.

**Figure 1:**
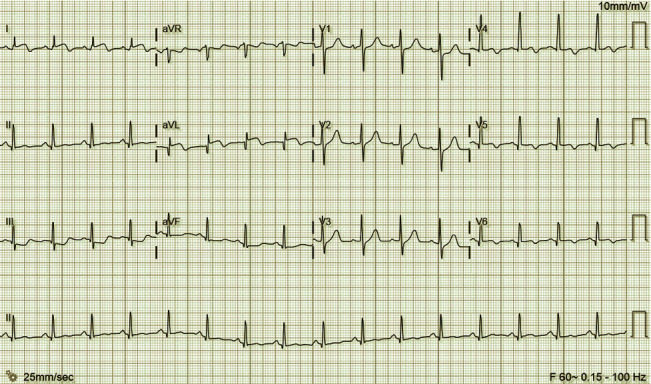
EKG at initial presentation to adult ED. Sinus rhythm with ST elevations in leads I and aVL, T-wave inversions in the inferior and lateral leads, and prolonged QT interval.

On presentation, he was well appearing. Vital signs were BP 107/56, heart rate 97, temperature 99.4°F, respiratory rate 18, and O_2_ saturation 95% on room air. Cardiac exam was unremarkable. Lungs were clear to auscultation bilaterally without wheezing, rhonchi, and crackles. There was no jugular venous distension, hepatomegaly, or pedal edema. Extremities were well perfused, and pulses were 2+ and symmetric throughout. Complete metabolic panel and blood count with differential were unremarkable. A drug toxicology panel screening for nine drugs, which tested for cocaine, amphetamines, etc., was completely negative. EKG demonstrated ST elevations in leads I and aVL and *T*-wave inversions in the inferior and lateral leads (Figure 1). Additional clinical laboratory tests revealed elevated cardiac and inflammatory markers (Table 1). Given the patient's age, both adult and pediatric cardiology were consulted; further management decisions were made jointly between these teams. A bedside transthoracic echocardiogram revealed regional wall motion abnormalities in the inferior left ventricular (LV) segments with preserved global LV systolic function.

**Figure 2:**
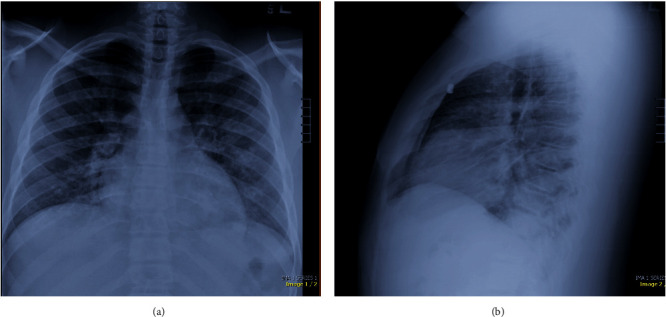
Chest X-ray at presentation with PA (a) and lateral (b) views with scattered hazy opacities and no confluent consolidation, pulmonary edema, pleural effusions, or pneumothorax. The cardiac silhouette is within normal limits. Findings are suggestive of an acute inflammatory process.

**Figure 3:**
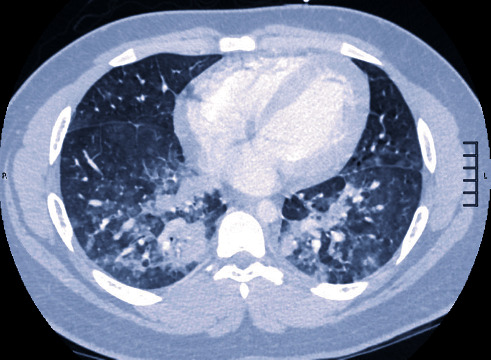
Chest CTA (lung window) at presentation showing multifocal consolidated and ground-glass opacities in the bilateral lungs.

Two hours into his presentation, the patient became hypoxic to 91% on pulse oximetry and was placed on 4L nasal cannula 100% FiO_2_ with improvement in O_2_ saturations to 96%. Chest X-ray (CXR) showed scattered bilateral hazy opacities (Figure 2). These additional clinical changes prompted a computed tomographic angiogram (CTA) to rule out pulmonary embolism (PE). Chest CTA was notable for multifocal consolidated ground-glass opacities in the lungs bilaterally (Figure 3). These imaging findings raised a significant concern for COVID-19. The decision was made to not pursue cardiac catheterization at this time.

He was transferred to the pediatric ED and subsequently admitted to the Pediatric Intensive Care Unit (PICU) for further evaluation with differential diagnosis including COVID-19-related myocarditis, non-COVID-19 myocarditis, and MIS-C. The patient had not received any doses of any COVID-19 vaccines. Further laboratory studies were notable for negative SARS-CoV-2 RNA and elevated levels of ESR (68 mm/hr) and CRP (201.3 mg/L) (Table 1). Formal echocardiogram performed the next morning revealed mild-to-moderate mitral valve regurgitation, low-to-normal left ventricular systolic function with an ejection fraction of 52%, regional wall motion abnormalities of the inferolateral LV wall, and normal origin of both coronary arteries with no dilation or aneurysms. Our institutional multidisciplinary MIS-C protocol was activated. Our patient was treated with 1 mg/kg methylprednisolone every 12 hours and heparin while undergoing evaluation for MIS-C. IVIg was not given due to the patient's depressed cardiac function, mild disease, and normal coronary arteries on the echocardiogram. Our institutional protocol recommends steroids and anticoagulation for MIS-C with addition of IVIg if there is clinically moderate-to-severe disease or if criteria are met for Kawasaki Disease (including coronary artery abnormalities). The LIAISON^®^ SARS-CoV-2 S1/S2 IgG test was conducted to detect IgG against SARS-CoV-2; this test has been found to have a false positive rate of up to 1.1% and a false negative rate of up to 1.9% [5]. SARS-CoV-2 IgG and repeat PCR were subsequently negative.

The patient remained afebrile throughout his hospitalization; as fever is a key manifestation of MIS-C, this further made a diagnosis of MIS-C unlikely. At this time, non-COVID-19 myocarditis was thought to be the most likely diagnosis and further work up was pursued in that direction. His work up was negative for autoimmune etiologies of myocarditis, including systemic lupus erythematosus and sarcoidosis, as well as for viruses commonly known to cause myocarditis: adenovirus, herpesviruses (cytomegalovirus, Epstein–Barr virus, and HHV-6), and parvovirus B19. Another pathogen associated with myocarditis is *Mycoplasma pneumoniae*, which the patient tested negative for by PCR, as well.

**Figure 4:**
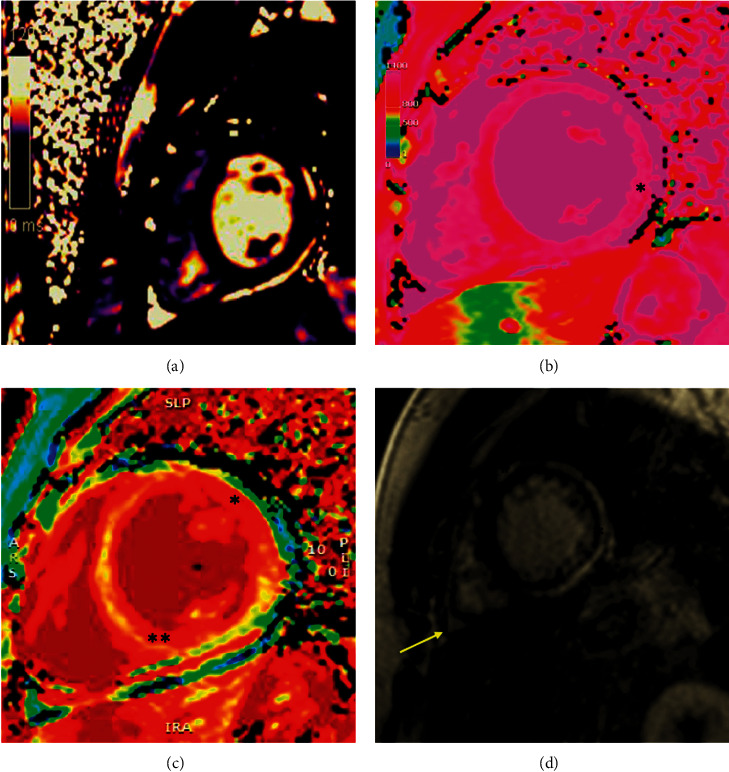
Parametric mapping and tissue characterization in myocarditis on cardiac MRI. (a) Short-axis midmyocardial T2 map with normal relaxation time and no evidence of acute edema. (b) Precontrast native midmyocardial T1 map showing diffusely elevated T1 relaxation time specifically along the lateral wall (^*∗*^). (c) Midmyocardial extracellular volume (ECV) map indicating increased ECV along the anterior and lateral walls (^*∗*^) and the inferoseptal wall (^*∗∗*^). (d) Postcontrast pulse sequence inversion recovery image of the midmyocardium with increased pericardial signal (^*∗∗*^), evidence of diffuse subepicardial late gadolinium enhancement along the entirety of the lateral wall (^*∗*^), and mild pericardial effusion (arrow).

A cardiac MRI was performed to assess the diagnostic MRI criteria for myocarditis. Cardiac MRI is considered the gold standard noninvasive modality for diagnosis of myocarditis. His MRI was positive for subacute myopericarditis demonstrating patchy and extensive subepicardial and transmural myocardial late gadolinium enhancement of the LV lateral and inferior walls extending from the base to midventricle, along with enhancement of the pericardium with normal T2 relaxation times (Figure 4). Between hospital days 2 and 3, he was weaned off supplemental oxygen. Cardiac and inflammatory markers gradually improved (Table 1). The day before discharge, he developed new asymptomatic, isolated premature ventricular contractions without sustained arrhythmias. He was discharged home with a diagnosis of myopericarditis of unclear etiology on a prednisone taper. At 8-week follow-up, he remained clinically asymptomatic. Repeat cardiac MRI revealed persistent, extensive fibrosis in different LV segments, similar to his initial MRI. Based on the American Heart Association myocarditis guidelines for return to competitive sports, he is currently restricted for at least 3–6 months, pending follow-up testing [6].

## 3. Discussion

Myocarditis has a broad spectrum of clinical presentations, ranging from asymptomatic to acute fulminant disease and sudden cardiac death, with continuously evolving diagnostic criteria; this makes the diagnosis quite challenging [3]. Pediatric myocarditis is considered a rare disease that accounts for about 0.05% of pediatric hospital discharges [4]. However, given its variable, often subclinical presentation, its true prevalence remains unknown and may be greater than documented [1]. Retrospective cohort studies have delineated a bimodal distribution for acute myocarditis in the pediatric population with most cases occurring in infants and adolescents [1,7].

Classically, myocarditis presents with a preceding viral prodrome. Many viruses are recognized as the underlying etiology with enteroviruses (e.g., coxsackieviruses A and B), parvovirus B19, and human herpesvirus 6 (HHV-6) being most common in the current era [3,8,9]. Myocarditis and associated symptoms can also be caused by substances such as cocaine and amphetamines, for which our patient tested negative [3,8]. Resting tachycardia, ventricular arrhythmias, chest pain, respiratory distress, abdominal pain, and vomiting are common presenting symptoms of pediatric viral myocarditis [2,8,9]. Chest pain has been found to be a typical symptom of acute myocarditis in adolescents [10]. ECG abnormalities are found in approximately 90% of children with myocarditis: nonspecific ST-T wave abnormalities, ST-segment elevation, low-voltage QRS complexes, and atrioventricular conduction delays [2,8,9]. Cardiac biomarker abnormalities, such as troponin *T* and I, are commonly elevated in children with myocarditis [2,8]. Elevated troponin *T* levels have been observed in up to 65% of pediatric myocarditis cases [2]. Importantly, these ST changes can mimic acute MI, as in the case of our patient, making diagnosis difficult and prone to cognitive bias [3,10]. Reports have shown that cases of adolescents initially diagnosed with acute MI may have been myocarditis with an infarct-like presentation [10,11]. Though acute MI is extremely rare among adolescents, it has been described in association with the anomalous origin of coronary arteries [10,11].

The differential diagnosis based on presentation with respiratory symptoms, CXR findings, EKG abnormalities and elevated troponin level was broad including PE, myocarditis, and acute MI. However, during the COVID-19 pandemic, the differential for this patient's presentation would be incomplete without COVID-19-related myocarditis and MIS-C. Key diagnostic features of myocarditis, MI, MIS-C, and COVID-19-related myocarditis are summarized (Table 2). CTA in our case helped rule out the anomalous coronary artery origin, which would be the most likely cause for MI in a young adult, and PE. The diagnostic challenge was then refocused on differentiating COVID-19-related cardiac disease versus non-COVID-19-related myocarditis.

Myocardial injury defined by elevated cardiac biomarkers has been reported in multiple reviews of adult patients hospitalized with COVID-19 [18–20]. Myocardial injury is rare in children with acute COVID-19 compared to adults; however, children with MIS-C can have significant myocardial involvement [13–15,21]. The understanding of MIS-C is still evolving. Cardiac findings of MIS-C encountered in the pediatric population include myocardial dysfunction, acute myocarditis or myocarditis-like clinical picture, and coronary artery dilation or aneurysms [21–23]. Dufort et al. reported that prevalence of myocarditis was the highest among adolescents, relative to other pediatric age groups, with MIS-C [23]. In the pediatric population, case series and case reports describing myocarditis in the setting of COVID-19 and MIS-C have been recently published; these patients tended to be critically ill, presenting with severe abdominal pain, vomiting, and fever [24–26].

Cardiac MRI is considered the gold standard noninvasive modality for diagnosis of myocarditis. We are still learning about the cardiac changes on MRI in patients with MIS-C. One published case series found evidence of myocardial injury on cardiac MRI in pediatric patients with MIS-C to be dissimilar to those with myocarditis in the acute phase; in this series, cardiac MRI in patients with MIS-C showed diffuse myocardial edema without evidence of focal late gadolinium enhancement [27]. On echocardiography, systolic dysfunction can be seen in both myocarditis and MIS-C [16]. However, in MIS-C, diastolic dysfunction has been shown to persist even after systolic dysfunction resolves [16]. Our patient did not have any evidence of diastolic dysfunction.

Most recently, with the approval of the Pfizer-BioNTech COVID-19 vaccine for children 12 years of age and older in the U. S., there have been rare cases of myocarditis and myopericarditis reported in adolescent males within 4 days of receiving the second vaccine dose [28]. The seven adolescent males presented with chest pain and evidence of myocarditis on cardiac MRI [28]. This series of cases presents an additional layer of complexity to the diagnosis of pediatric myocarditis in the current era. Of note, our patient presented prior to the approval and availability of the Pfizer-BioNTech COVID-19 vaccine and, therefore, had not received any doses. Though a causal relationship between the vaccine and myocarditis has not yet been established in the pediatric population, healthcare providers' awareness of myocarditis as a possibility after COVID-19 vaccination is crucial for appropriate referral to pediatric cardiology and further management. This awareness may also spare otherwise healthy adolescents presenting with chest pain, like our patient, from invasive procedures such as cardiac catheterization.

The management of acute myocarditis is mainly supportive [3]. The use of IVIg, which has antiviral, anti-inflammatory, and immunomodulatory effects, remains controversial; IVIg has been shown to provide meaningful benefit in some pediatric patients, though not definitively [3,8,29]. The role of steroids for treatment of myocarditis also remains controversial [29]. Despite this controversy, prednisone is used in about 25–30% of acute myocarditis cases in the United States [3,8]. In MIS-C, there is some evidence suggesting that combination therapy with IVIg and steroids is associated with reduction in recovery time of LV systolic function and reduced ICU stay [14]. Currently, though there are broad guidelines and expert consensus, management of MIS-C and myocarditis remains dependent on institutional protocols.

Acute pediatric myocarditis is often associated with heart failure [3]. Heart failure in the setting of adult myocarditis is managed with diuretics, angiotensin‐converting enzyme inhibitors, or angiotensin receptor blockers and *β*‐blockers. However, in pediatrics, a conventional heart failure regimen has not yet been established [8]. Many pediatric myocarditis patients receive supportive care in an ICU at presentation, like our patient. Inotropic agents are used with or without extracorporeal membrane oxygenation for patients with cardiogenic shock or patients who deteriorate despite medical treatment [3,8]. Pediatric myocarditis can deteriorate into fulminant myocarditis and continues to have significant morbidity and mortality [1,9]. Thus, myocarditis is a must-not-miss diagnosis that is important to recognize, treat, and monitor closely with serial echocardiograms.

The current case highlights how challenging the diagnosis of myocarditis can be in the pediatric population, especially in the era of COVID-19. Adolescents are a unique cohort where collaboration between pediatric and adult specialists is essential for optimal clinical care. With this case, we illustrate a wide range of differential diagnoses that have overlapping features of classic myocarditis. As our knowledge of the cardiac manifestations of COVID-19 and MIS-C evolves, it is crucial that myocarditis continues to be part of clinicians' diagnostic thinking.

## Figures and Tables

**Table 1:** Laboratory values from the time of presentation and at the time of discharge.

	Initial presentation	At discharge
Troponin T	1.11 ng/mL (normal reference <0.01)	0.10 ng/mL
N-terminal pro-B-type natriuretic peptide (NT-proBNP)	2,312 pg/mL (<125)	796 pg/mL
D-dimer	2.29 mg/L FEU (<0.50)	0.73 mg/L FEU
Fibrinogen	851 mg/dL (194–448)	
CRP	201.3 mg/L^a^	8.2 mg/L
ESR	68 mm/hr (0–20)	12 mm/hr^b^
Ferritin	121 ng/mL (30–400)	51 ng/mL
Angiotensin-converting enzyme	31 U/L (8–52)	20 U/L
Interleukin-6	65.2 pg/mL (<5)	-

^a^CRP <1.0 indicates lower relative cardiovascular (CV) risk, 1.0–3.0 = average relative CV risk, 3.0–10.0 = higher relative CV risk, and >10.0 may be associated with infection and inflammation. ^b^ESR value is from follow-up appointment, which was 3 days after discharge.

**Table 2:** Comparison of key features of pediatric myocarditis, myocardial infarction, MIS-C (focusing on cardiovascular manifestations), and COVID-19-related myocarditis.

	Acute myocarditis [8–10]	Acute myocardial infarction [10–12]	MIS-C [13–16]	COVID-19-related myocarditis^*∗*^ [17]
Fever	May be present	Unlikely	Persistent	Common
Clinical symptoms	Resting tachycardia, chest pain, palpitations, shortness of breath, respiratory distress, abdominal pain, and vomiting	Can present with chest pain	Multisystem involvement, including rash, tachycardia, abdominal pain, vomiting, diarrhea, and conjunctivitis	Shortness of breath, respiratory distress, chest pain, abdominal pain, vomiting, diarrhea, etc.
Cardiac biomarkers (troponin I or T)	Elevated	Elevated	Elevated (mild to moderate)	Elevated
Inflammatory markers (CRP, ESR, ferritin, etc.)	Often elevated	Normal	Elevated	Elevated
Echocardiogram findings	Regional wall motion abnormalities; decreased LV systolic function	Coronary arteries may be normal or anomalous	Coronary artery dilatation; systolic dysfunction that resolves; and persistent diastolic dysfunction	Reduced LV ejection fraction and pericardial effusion

^*∗*^COVID-19-related myocarditis features include data from adults.

## Data Availability

The literature review data used to support the findings of this study are included within the article. I provide consent and attest to my contributions.
